# A Thematic Analysis Service Evaluation of the 'Mind, Body Connection' (Yoga, Meditation and Mindfulness Course) Within a Borderline Personality Disorder Therapeutic Community.

**DOI:** 10.1192/bjo.2026.11580

**Published:** 2026-06-30

**Authors:** Isabella Walker, Simon Graham, Samuel Button

**Affiliations:** 1 NHS Lothian, Edinburgh, United Kingdom; 2 Merseycare, Liverpool, United Kingdom

## Abstract

**Aims:**

Mindfulness, Meditation and Yoga have an evidence base for improving emotional regulation – with little research looking at the impact on a Borderline Personality Disorder (BPD) cohort. We investigated whether a supplementary session programme could improve emotional regulation and thus symptomatology in BPD service users.

Our initial objective is to identify whether there is an evidence base to support the need for a ‘Mind, body connection’ programme. The subsequent objective is to further develop and improve the ‘Mind, body connection’ programme in response to feedback from the service users.

AIM 1. To establish whether there is benefit from a ‘Mind, Body connection programme’.

AIM 2. To establish further understanding of the holistic effects of the ‘Mind, body connection’ programme for service users.

Future AIM 1. To identify direct benefits of Yoga, Mindfulness and Meditation in BPD populations.

Future AIM 2. To develop a science backed programme to provide a supportive therapeutic practice for BPD patients.

**Methods:**

A Thematic analysis was conducted to investigate the ‘Mind, Body Connection’ sessions with service users attending a bi-weekly Therapeutic Community at the Personality Disorder Hub. A 50 minute tape-recorded, in-depth, semi-structured interview took place which was then transcribed to allow for coding.

**Results:**

The theme of 'Greater Capacity to stay present' summarises how greater 'emotional regulation' and 'body awareness' can develop. Both ‘body awareness’ and emotional regulation’ proved to be double edged swords. By developing greater awareness of how your body holds emotions, participants reported greater ability to focus on the body and stay in control of emotions, but also a greater awareness of pain. Service users oppositions - transitioning from a state of doing to a state of being and difficulty around ‘self-dismissing’. Results were bidirectional - higher levels of emotional dysregulation impaired mindfulness practice but increased frequencies of mindfulness practice to >3 times a week was statistically significant in reducing emotional dysregulation symptoms.

**Conclusion:**

The ‘Mind, Body Connection’ programme has a positive impact on service users – but significant support should be provided for its associated challenges. Recommendations; increasing the frequency of Yoga, Mindfulness and Meditation practice to>3 times per week, at home resources and guidance change to the management of BPD (development of patient informed supplementary sessions). Practice in the present underscores the ability to stay present in higher states of arousal in the future. Further research should investigate the process of ‘self-dismissal’ as a regulation adjacent skill.

